# Does the cardiovascular drug levosimendan prevent iodinated contrast medium nephrotoxicity with glycerol aggravation in rats?

**DOI:** 10.1186/s41747-021-00249-7

**Published:** 2021-11-17

**Authors:** Irmak Durur-Subasi, Duygu Kose, Muhammed Yayla, Busra Sirin, Adem Karaman, Ilknur Calik, Fatih Alper

**Affiliations:** 1 Department of Radiology, International Faculty of Medicine, Istanbul Medipol University, TEM Avrupa Otoyolu, Goztepe Cikisi No:1, Bagcilar, Istanbul, Turkey; 2 Department of Radiology, Ataturk University Faculty of Medicine, Erzurum, Turkey; 3 Department of Pharmacology, Faculty of Medicine, Ataturk University, Erzurum, Turkey; 4 Department of Pharmacology, Kafkas University Faculty of Medicine, Kars, Turkey; 5 Department of Pathology, Faculty of Medicine, Fırat University, Elazig, Turkey

**Keywords:** Contrast media, Drug-related side effects and adverse reactions, Iohexol, Levosimendan, Rats (Wistar)

## Abstract

**Background:**

We investigated whether levosimendan prevents contrast medium nephrotoxicity with glycerol aggravation in rats.

**Methods:**

Forty-eight Wistar albino rats were assigned to eight groups (*n* = 6 × 8). No medication was administered to group I (controls); glycerol (intramuscular injection of 25% glycerol, 10 mL/kg) group II; intravenous iohexol 10 mL/kg to group III; glycerol and iohexol to group IV; iohexol and intraperitoneal levosimendan 0.25 mg/kg to group V; glycerol, iohexol, and levosimendan 0.25 mg/kg to group VI; iohexol and levosimendan 0.5 mg/kg to group VII; and glycerol, iohexol, and levosimendan 0.5 mg/kg to group VIII. One-day water withdrawal and glycerol injection prompted renal damage; iohexol encouraged nephrotoxicity; levosimendan was administered 30 min after glycerol injection and continued on days 2, 3, and 4. The experiment was completed on day 5. Serum blood urea nitrogen (BUN) and creatinine levels, superoxide dismutase (SOD) activity, glutathione (GSH), malondialdehyde (MDA) levels, tumour necrosis factor-α (TNF-α), nuclear factor kappa ß (NFK-ß), interleukin 6 (IL-6), and histopathological marks were assessed. One-way analysis of variance and Duncan’s multiple comparison tests were used.

**Results:**

Levosimendan changed serum BUN (*p* = 0.012) and creatinine (*p* = 0.018), SOD (*p* = 0.026), GSH (*p* = 0.012), and MDA (*p* = 0.011). Levosimendan significantly downregulated TNF-α (*p* = 0.022), NFK-ß (*p* = 0.008), and IL-6 (*p* = 0.033). Histopathological marks of hyaline and haemorrhagic cast were improved in levosimendan-injected groups.

**Conclusion:**

Levosimendan showed nephroprotective properties due to its vasodilator, oxidative distress decreasing and inflammatory cytokine preventing belongings.

## Key points

- Levosimendan diminished blood urea nitrogen and creatinine levels in contrast medium nephrotoxicity aggravated by glycerol.
- Superoxide dismutase activity and glutathione levels were ameliorated by levosimendan in contrast medium nephrotoxicity with glycerol aggravation.
- Levosimendan diminished malondialdehyde levels in contrast medium nephrotoxicity.
- Tumour necrosis factor-α, nuclear factor kappa ß, and interleukin 6 were decreased by levosimendan in contrast medium nephrotoxicity.
- Levosimendan ameliorated the histopathological score of rat kidney in contrast medium nephrotoxicity.

## Background

Contrast medium nephrotoxicity is defined as a deterioration in kidney function arising within three days after the parenteral injection of a contrast medium in the absence of any additional cause [1, 2]. Contrast medium nephrotoxicity is responsible for 12% of all acute renal failure in hospitalised patients [3].

Three paths suggested to explain the pathophysiology of contrast medium nephrotoxicity are direct tubular cell toxicity of the contrast medium, haemodynamic effects, and biochemical disturbances (free radicals and antioxidant enzyme activity). The inflammatory process contributes to the nephrotoxicity effects with pro-inflammatory mediators due to the damage of tubular-endothelial cells and inflammatory cytokines further contributing to the growing kidney damage [4, 5]. Therefore, experimental animal model may help to better understand the physio-pathological mechanism and to develop treatments to prevent the occurrence of contrast medium nephrotoxicity.

While some drugs increase the risk of contrast medium nephrotoxicity, research continues on the potential protective effects, too [6–9]. Several researches are aimed to investigate the nephroprotective effect of some drugs, in particular aminophylline, statins, vitamin C, and iloprost, but further evaluation is needed [8]. Levosimendan is a drug that was developed for acute heart failure. It increases calcium sensitivity and opens adenosine triphosphate-dependent potassium channels. Its positive inotropic, vasodilator, and cardio-protective effects have been widely explored. It was suggested that levosimendan can protect the pulmonary, gastrointestinal, and nervous systems; the vascular endothelium; the liver; and the kidneys [10]. Additionally, levosimendan attenuates inflammatory gene expression in endothelial cells, while other recent studies showed that levosimendan has anti-inflammatory activity and modulates pro-inflammatory cytokines, as well as decreasing oxidative distress [11, 12]. There is no study on the kidney-sparing role of levosimendan for contrast medium nephrotoxicity exacerbated by glycerol.

In this experimental study, we targeted to study the nephroprotective role of levosimendan using biochemical, molecular, and histopathological analyses in rats afflicted by contrast medium nephrotoxicity with the glycerol-induced renal functional disorder.

## Methods

### Ethical approval

The Institutional Review and Animal-Care Boards of Ataturk University Faculty of Medicine approved the study. All the investigations and procedure were made in agreement with nationwide guiding principles on behalf of the practice and research animal care. The study was blinded. The conduct of experiments (DK, IDS, AK), outcome assessment (MY), and data analyses (BS, IC, FA) were independently done.

### Experimental design

**Fig. 1:**
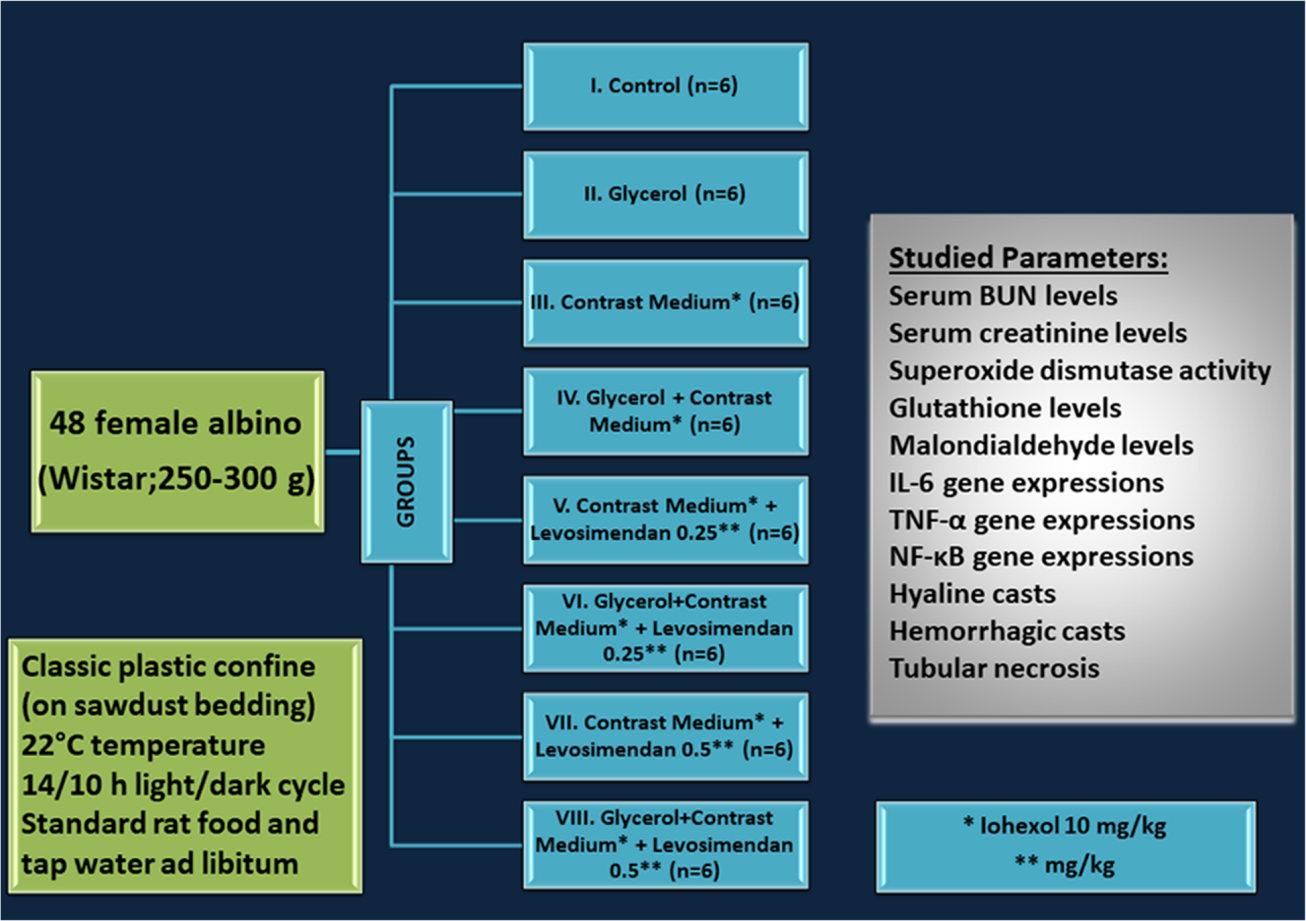
Forty-eight female albino Wistar rats (250–300 g) were enrolled. Eight groups were made of six rats in each confine. The rats were housed in standard plastic cages on sawdust bedding in an air-conditioned room at 22°C under controlled light conditions (14/10 h light/dark cycle). Standard rat chow and tap water were provided *ad libitum*

Forty-eight female Wistar albinos were enrolled those were gained from our Research Laboratory. They were 3–4 months old, 250–300 g, and healthy and never took part in any other experiment before. The groups had been created as shown in Fig. 1 at the stated conditions. The cages (classic plastic confine on sawdust bedding), temperature (22 °C), light (14/10 h light/dark cycle), and feeding (standard rat food and tap water *ad libitum*) were controlled and uniformity was ensured.

A 1-day water withdrawal and intramuscular injection of 25% glycerol (Bikar Medical Products, Istanbul, Turkey) prompted renal damage in groups of II, IV, VI, and VIII with a dosage of 10 ml/kg to gluteal muscle. Twenty-four hours after glycerol injection, intravenous delivery of 10 ml/kg iohexol (Omnipaque, Opakim Medical Products, Istanbul, Turkey) via tail vein stimulated nephrotoxicity. Thirty minutes after glycerol injection, levosimendan was injected intraperitoneally to the V, VI, VII, and VIII groups. Levosimendan administration was continued for the second, third, and the fourth days.

The experiment was completed on the 5th day of glycerol injection by the high dose of thiopental sodium (50mg/kg). Under general anaesthesia, blood specimens were taken from the cardiac cavity. Kidneys were excised straightaway for biochemical evaluations, molecular analyses, and pathological examination.

### Blood urea nitrogen, creatinine, and biochemical analyses

Serum blood urea nitrogen (BUN) (Lot number B0382A, S.r.i, Italy) and creatinine (Lot number B0914A, S.r.i, Italy) were identified using commercially existing supplies. Analyses were done in a ChemWell 2910-automated-EIA and chemistry analyser (Awareness Technology, Inc., Palm City, FL, USA).

After surgical excision, the kidneys were stored at −80 °C. The tissues were initially perfused by phosphate-buffered saline (PBS)/heparin and then ground in liquid nitrogen using the TissueLyser II grinding jar set (Qiagen, Hilden, Germany). Approximately 100 mg of ground tissue was homogenised in 1 mL PBS homogenate buffer in an Eppendorf tube with TissueLyser II, and the samples were then centrifuged. Superoxide dismutase (SOD) activity, glutathione (GSH), and malondialdehyde (MDA) levels from supernatants and standards were measured at room temperature in duplicate via modified methods, with an enzyme-linked immunosorbent assay, ELISA, reader. A standard curve was plotted and the equation was obtained from the absorbance of the standards. The linear SOD, GSH, and MDA concentrations were calculated according to this equation and were expressed as U/mg-protein, nmol/mg-protein, and nmol/mg-protein, respectively. The data obtained were presented as mean ± standard deviation as 1-mg protein.

### Molecular analyses

#### Total ribonucleic acid (RNA) extraction and complementary deoxyribonucleic acid (cDNA) synthesis

The tissues (20 mg) were stabilised in RNA stabilisation reagent (RNAlater, Qiagen, Hilden, Germany), and then disrupted using the TissueLyser II (Qiagen, Hilden, Germany). Total RNA was purified using the RNeasy Mini Kit (Qiagen, Hilden, Germany) in a QIAcube (Qiagen, Hilden, Germany), based on the manufacturer’s instructions. The RNA samples were then reverse-transcribed into cDNA: using the High Capacity cDNA Reverse Transcription Kit (Applied Biosystems, Foster City, CA). From 10 μL, total RNA was treated with 2 μL 10X RT buffer, 0.8 μL 25X dNTP mix, 2 μL 10X RT random primers, 1 μL multiscribe reverse transcriptase, and 4.2 μL DEPC-H2O. Reverse transcription was conducted at 25 °C for 10 min, followed by 120 min at 37 °C, and finally, 5 min at 85 °C, using a Veriti 96-well thermal cycler (Applied Biosystems, Foster City, CA, USA). The concentration and quality of the cDNA were assessed and quantified using the Epoch Spectrophotometer System and Take3 Plate (BioTek Inc., Winooski, VT, USA).

#### Relative quantification of gene expression

Relative tumour necrosis factor-α (TNF-α), nuclear factor kappa beta (NFK-ß), and interleukin-6 (IL-6) messenger RNA (mRNA) expression analyses were performed using StepOne Plus Real-Time polymerase chain reaction (PCR) system technology (Applied Biosystems, Foster City, CA, USA) using synthesised cDNA from rat kidney RNA. A quantitative PCR was run using a TaqMan probe mix based on TaqMan probe-based technology (Applied Biosystems, Foster City, CA, USA). Real-time PCR was performed using primers generated for rat TNF-α Rn00562055_m1, rat NFK-ß Rn01399583_m1, rat IL-6 Rn01410330_m1, and rat β-actin Rn00667869_m1. The results are expressed as relative-fold, compared with control animals. The expression data for β-actin in each tissue were used as the endogenous control. Each determination was performed in triplicate for each tissue in a 96-well optical plate for both targets, using 9-μL cDNA (100 ng), 1 μL of Primer Perfect Probe mix, and 10 μL of QuantiTect Probe PCR Master Mix (Qiagen) in each 20-μL reaction. The plates were heated for 2 min at 50 °C and then 10 min at 95 °C. Subsequently, 40 cycles of 15 s at 94 °C and cycles of 60 s at 60 °C were conducted. All data are expressed as the fold-change in expression compared with the expression in other animal groups, using the 2-delta-delta Ct (2-ΔΔCt) method [13, 14].

### Pathologic analyses

Kidneys of the rats in all groups were obtained, sectioned in coronal plane, and fixed at 10% neutral formalin for 48–72 h. The tissues were then routinely processed, and embedded in paraffin wax, and 4–5-μm thick serial sections were cut. All tissue sections were stained with haematoxylin and eosin for histopathological assessment and examined under a light microscope (Olympus BX51, Tokyo, Japan). Hyaline and haemorrhagic casts and tubular necrosis were evaluated and counted. A minimum of five fields for each kidney slide at 100× magnification was evaluated. The severity of the changes was evaluated using scores on the following scale: grade 0, negative; grade +1, mild; grade +2, moderate; grade +3, severe; and grade +4, most severe [14].

### Statistical analysis

Statistical analysis was performed using SPSS 20.0 software (IBM Corp. SPSS Statistics for Windows, Armonk, NY, USA). Continuous variables are displayed as the mean ± standard deviation, and categorical variables are reported as counts and percentages. Comparisons of biochemical evaluations and molecular analyses among groups were performed using a one-way analysis of variance and Duncan’s multiple comparison tests. A *p*-value under 0.05 was set as statistically significant.

## Results

### Blood urea nitrogen, creatinine, and biochemical analyses

**Fig. 2:**
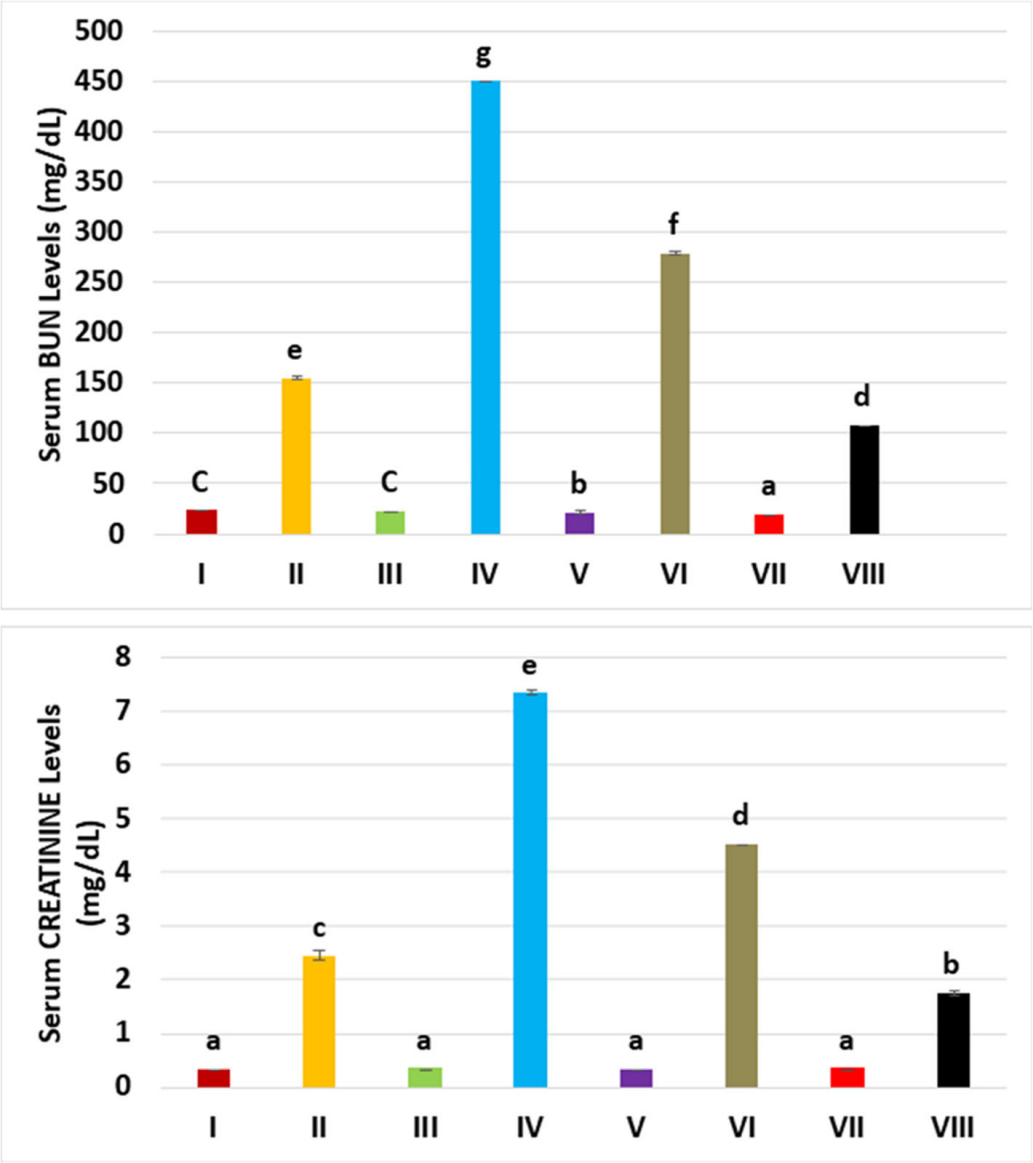
The mean blood urea nitrogen (BUN) and creatinine levels for the experimental groups. Mean BUN level is the highest in group IV (glycerol plus contrast medium) and levosimendan administration lowered the levels of BUN. Serum creatinine levels showed similar trends with the administered drugs. Means in the same column with the same letter are not significantly different; means in the same column with different letters indicate the statistically significant differences between the groups.

BUN levels showed statistically significant difference between the groups (*p* = 0.012). Similarly, creatinine levels showed statistically significant difference between the groups (*p* = 0.018). BUN and creatinine serum levels meaningfully higher in the glycerol and contrast medium groups (i.e., groups II, IV, VI, and VIII) versus controls and the other groups. However, levosimendan injection substantially reduced the levels of BUN and creatinine compared with glycerol and contrast medium group. Also, levosimendan at a high dose was more effective than at a low dose (Fig. 2, Table 1).

**Table 1:** The mean blood urea nitrogen (BUN) and creatinine levels according to experimental groups

	BUN (mg/dL)		Creatinine (mg/dL)	
Groups	Mean	Standard deviation	Mean	Standard deviation
Healthy (I)	23.285	1.500	0.325	0.019
GLY (II)	154.737	1.708	2.445	0.071
CM (III)	22.395	0.804	0.342	0.015
GLY+CM (IV)	451.245	0.768	7.340	0.029
CM+LEVO 0.25 (V)	21.130	0.747	0.333	0.012
GLY+CM+LEVO 0.25 (VI)	278.143	1.026	4.522	0.008
CM+LEVO 0.5 (VII)	18.995	0.520	0.337	0.027
GLY+CM+LEVO 0.5 (VIII)	107.913	0.722	1.750	0.033

*BUN* Blood urea nitrogen, *CM* Contrast medium, *GLY* Glycerol, *LEVO* Levosimendan

**Fig. 3:**
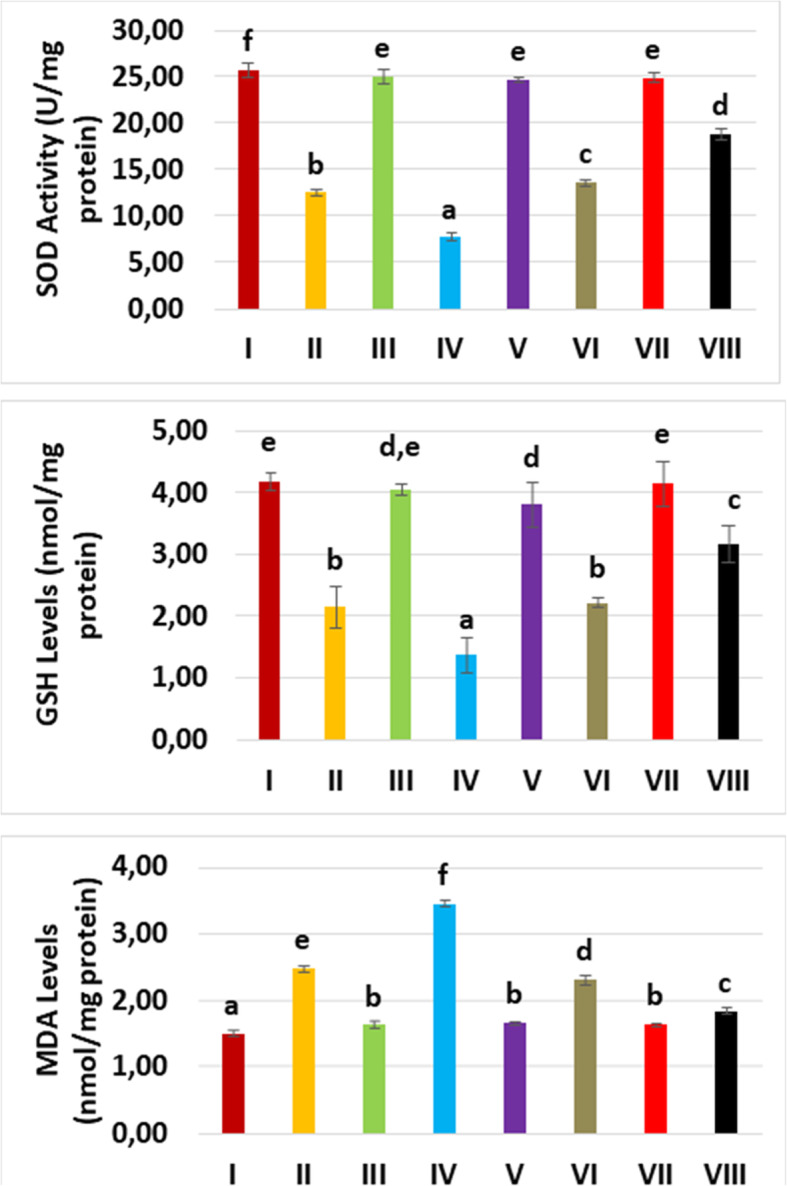
The superoxide dismutase (SOD) activity, glutathione (GSH), and malondialdehyde (MDA) levels of all experimental groups. Although the glycerol and contrast media administration compromised SOD and GSH, levosimendan promotes SOD and GSH. An opposite effect of glycerol, contrast media, and levosimendan has been experienced for MDA

SOD activity was statistically different among the groups (*p* = 0.026) and meaningfully reduced for glycerol and contrast medium-used-groups relative to the control group. For the levosimendan administration groups, SOD levels were considerably increased in both levosimendan doses, additionally, its activity significantly improved with high dose administration of levosimendan (Fig. 3, Table 2).

**Table 2:** The mean superoxide dismutase (SOD) activity, glutathione (GSH), and malondialdehyde (MDA) levels according to experimental groups

	SOD U/mg-protein		GSH nmol/mg-protein		MDA nmol/mg-protein	
Groups	Mean	Standard deviation	Mean	Standard deviation	Mean	Standard deviation
Healthy (I)	25.720	0.742	4.170	0.129	1.510	0.039
GLY (II)	12.520	0.343	2.160	0.337	2.490	0.052
CM (III)	25.080	0.720	4.060	0.083	1.630	0.033
GLY+CM (IV)	7.700	0.395	1.370	0.274	3.450	0.039
CM+LEVO 0.25 (V)	24.770	0.216	3.820	0.365	1.660	0.033
GLY+CM+LEVO 0.25 (VI)	13.600	0.405	2.210	0.063	2.310	0.068
CM+LEVO 0.5 (VII)	24.950	0.485	4.150	0.361	1.650	0.026
GLY+CM+LEVO 0.5 (VIII)	18.850	0.602	3.160	0.289	1.840	0.035

*CM* Contrast medium, *GLY* Glycerol, *GSH* Glutathione, *LEVO* Levosimendan, *MDA* Malondialdehyde, *SOD* Superoxide dismutase

GSH levels were statistically different among the groups (*p* = 0.012) and considerably reduced in the glycerol and contrast medium used groups compared to the control. GSH levels were considerably recovered in the levosimendan applied groups (Fig. 3, Table 2).

MDA levels were different statistically among the groups as shown in Fig. 3 and Table 2 (*p* = 0.011) and considerably augmented in glycerol and contrast medium used groups compared with the control group. The MDA levels were significantly diminished with the administration of levosimendan in both doses. Also, the analysis showed that the levosimendan at high dose was more effective than at the low dose.

### Molecular analyses

**Fig. 4:**
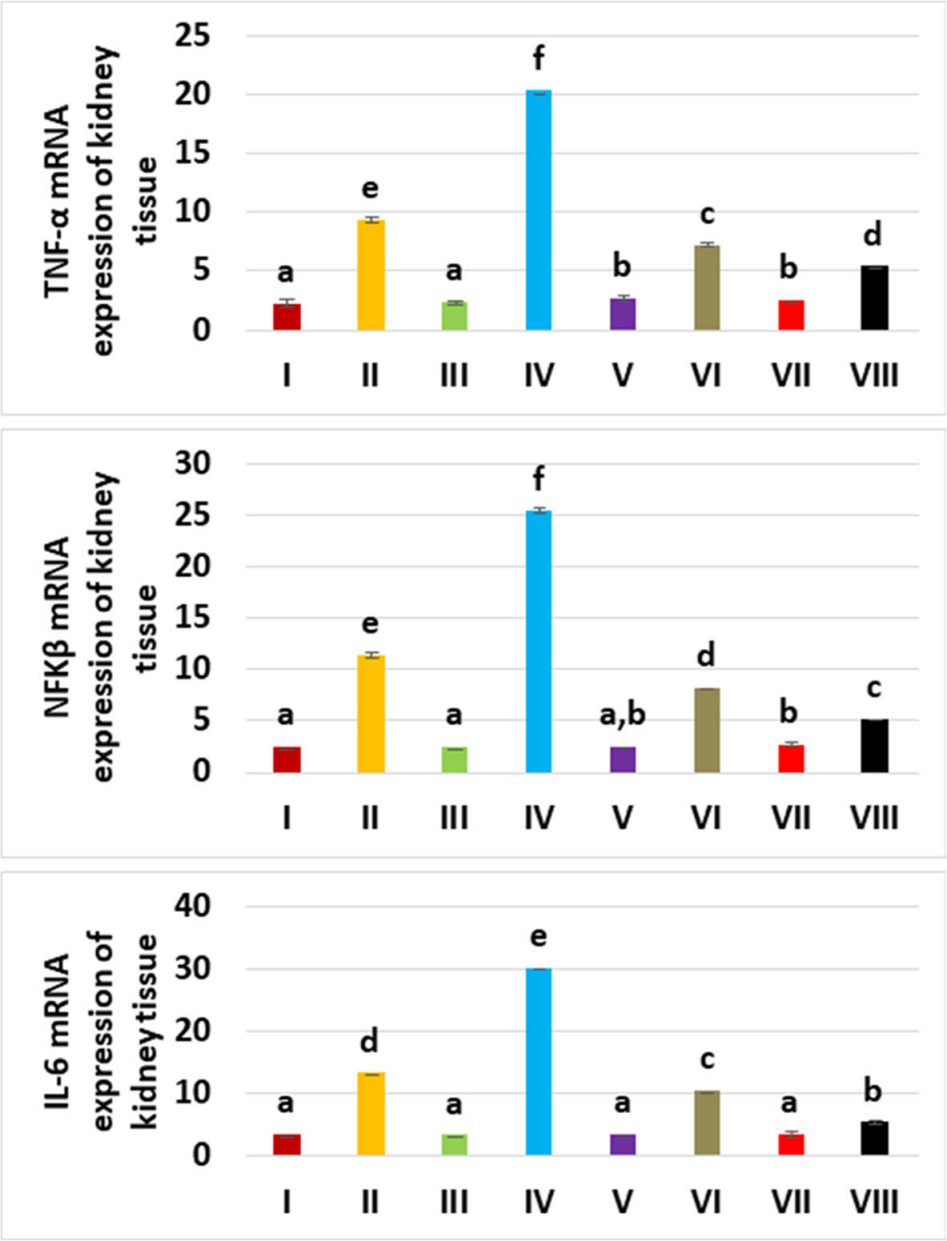
Tissue tumour necrosis factor-α (TNF-α), nuclear factor kappa β (NFK-β), and interleukin 6 (IL-6) mRNA expression of the experimental groups. TNF-α, NFK-β, and IL-6 mRNA expressions are promoted by glycerol and contrast media administration. However, levosimendan lowered the levels

TNF-α gene expressions were different among the groups (*p* = 0.022) and up-regulated in glycerol and contrast medium used groups (20.35-fold) compared to the control group (Fig. 4, Table 3). Administration of levosimendan produced a substantial down-regulatory consequence on TNF-α expression, by 7.16-fold for 0.25 mg/kg and 5.36-fold for 0.5 mg/kg of doses.

**Table 3:** The mean tumour necrosis factor α (TNF-α), nuclear factor kappa β (NFK-β), and interleukin 6 (IL-6) expressions according to experimental groups

	TNF-α (expressions relative to β-actin)		NFK-β (expressions relative to β-actin)		IL-6 (expressions relative to β-actin)	
Groups	Mean	Standard deviation	Mean	Standard deviation	Mean	Standard deviation
Healthy (I)	2.150	0.105	2.417	0.147	3.300	0.179
GLY (II)	9.317	0.232	11.333	0.216	13.250	0.188
CM (III)	2.300	0.141	2.450	0.164	3.200	0.110
GLY+CM (IV)	20.350	0.207	25.367	0.207	30.183	0.117
CM+LEVO 0.25 (V)	2.633	0.137	2.517	0.075	3.300	0.141
GLY+CM+LEVO 0.25 (VI)	7.167	0.103	8.167	0.103	10.333	0.216
CM+LEVO 0.5 (VII)	2.517	0.147	2.683	0.147	3.350	0.187
GLY+CM+LEVO 0.5 (VIII)	5.367	0.163	5.183	0.117	5.250	0.187

*CM* Contrast medium, *GLY* Glycerol, *IL-6* Interleukin 6, *LEVO* Levosimendan, *NFK-ß* Nuclear factor kappa ß, *TNF-α* Tumour necrosis factor α

NFK-ß expressions were different significantly among the groups (*p =* 0.008). As shown in Fig. 4, compared with the control group, the NFK-β mRNA level was considerably elevated in glycerol and contrast medium used groups by 25.36-fold. NFK-ß expression in both doses of levosimendan groups decreased in the rat kidney tissue. In the groups of V and VII, NFK-ß expression was respectively decreased to 8.16-fold and 5.18-fold compared to the groups of VI and VIII.

IL-6 mRNA levels were different statistically among the groups (*p =* 0.033). According to the control group, IL-6 mRNA levels were expressively induced in the glycerol and contrast medium used group, by 30.18-fold (Fig. 4). Levosimendan demonstrated a significant dose-dependent reducing influence on IL-6 mRNA expression, by 10.33 and 5.25-fold, respectively.

TNF-α, NFK-ß, and IL-6 were decreased prominently in the high dose levosimendan group (VIII) compared to group VI.

### Pathologic analyses

**Fig. 5:**
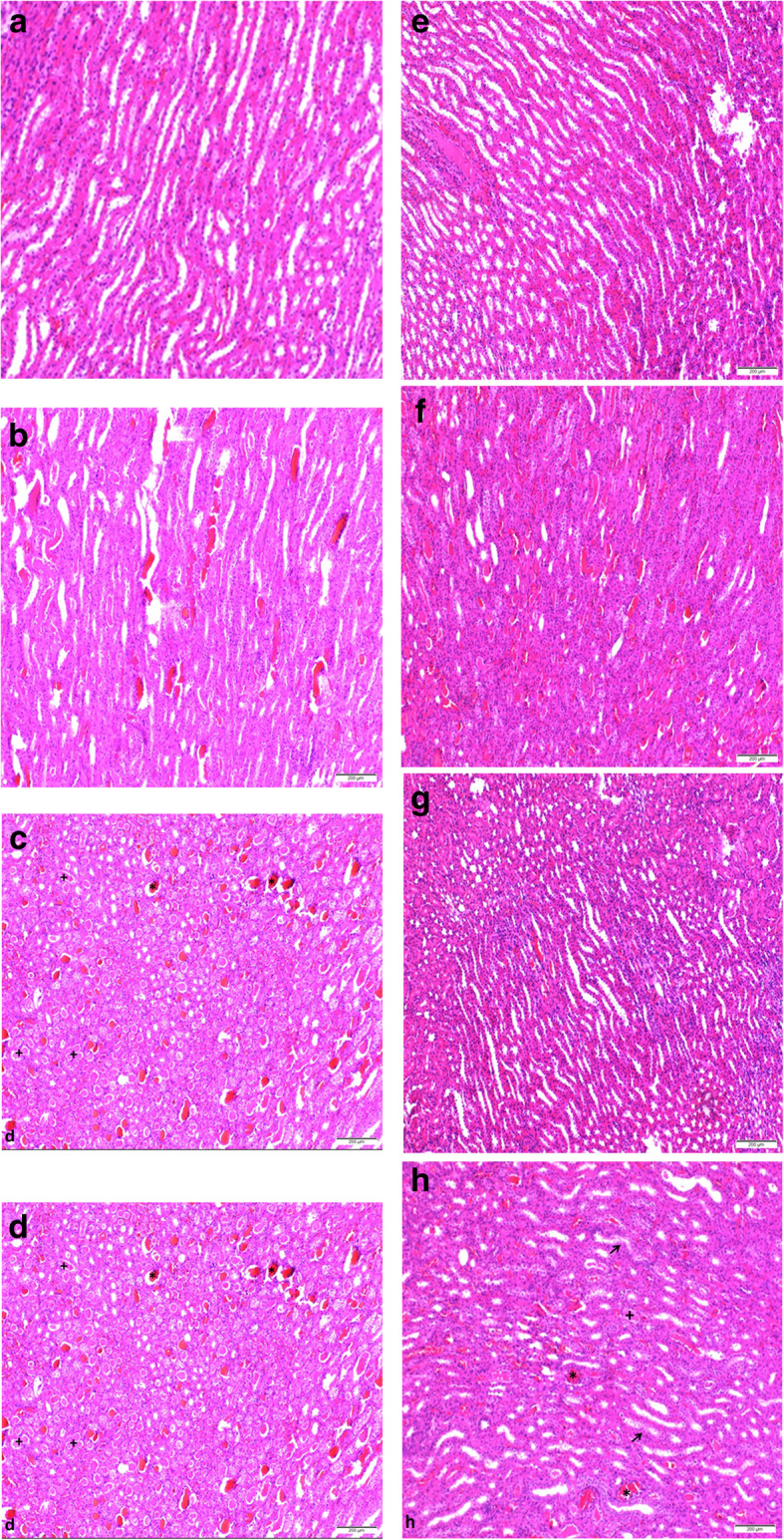
Haematoxylin and eosin result in rats’ kidney tissue; magnification 100×. Groups and histopathology specimens are seen. **a** Normal structure of kidney in the control group. **b** Hyaline and haemorrhagic casts are seen drastically and moderately; tubular necrosis also is present. **c** Only haemorrhagic casts are seen moderately; hyaline casts and tubular necrosis are observed slightly. **d** Hyaline (**+**) and haemorrhagic casts (*) are seen drastically; moderate tubular necrosis is present. **e** Only haemorrhagic casts are seen slightly. **f** Both hyaline casts and tubular necrosis are seen moderately; haemorrhagic casts are also present drastically. **g** Only haemorrhagic casts are seen slightly. **h** Hyaline (**+**) and haemorrhagic casts (*) are seen as decreased; tubular necrosis (arrow) is seen moderately

The histopathology results of renal tissues in all groups are shown in Table 4. The control group did not expose any pathological injury. Slight to drastic hyaline and haemorrhagic casts and tubular necrosis were detected in the groups, except for control group (Fig. 5). Levosimendan administration relieved the hyaline casts with both doses and haemorrhagic casts with high doses compared to glycerol and contrast medium groups.

**Table 4:** Histopathological scores according to experimental groups

Groups	Hyaline casts	Haemorrhagic casts	Tubular necrosis
Healthy (I)	Grade 0	Grade 0	Grade 0
GLY (II)	Grade 3	Grade 3	Grade 2
CM (III)	Grade 1	Grade 2	Grade 1
GLY+CM (IV)	Grade 3	Grade 3	Grade 2
CM+LEVO 0.25 (V)	Grade 0	Grade 1	Grade 0
GLY+CM+LEVO 0.25 (VI)	Grade 2	Grade 3	Grade 2
CM+LEVO 0.5 (VII)	Grade 0	Grade 1	Grade 0
GLY+CM+LEVO 0.5 (VIII)	Grade 1	Grade 1	Grade 2

*Grade 0* negative, *Grade 1* mild (+1), *Grade 2* moderate (+2), *Grade 3* severe (+3), *CM* Contrast medium, *GLY* Glycerol, *LEVO* Levosimendan

## Discussion

Our study showed that levosimendan administration significantly diminished serum levels of BUN and creatinine in contrast medium nephrotoxicity aggravated by glycerol. Changes in SOD activity, GSH, and MDA levels sustained anti-oxidative effects and the molecular results in terms of TNF-α, NFK-β, and IL-6 supported anti-inflammatory effects of levosimendan. Levosimendan administration improved the histopathological scores of nephrotoxic rats in terms of hyaline and haemorrhagic casts.

The pathogenesis of contrast medium nephrotoxicity is not known, but it was shown to be affected by toxic and ischemic injury to the renal tubular cells [14]. Contrast medium reduces the blood supply to the kidneys and causes hypoxia. Haemodynamic effects and cytotoxicity are the mechanisms responsible for the injury. In glycerol-induced renal failure, ischemic injury or tubular nephrotoxicity occurs [14]. Serum creatinine level is the most commonly used renal functional test together with blood urea nitrogen. Normally, renal glomeruli freely filter both urea and creatinine but in case of renal functional impairment the process is stopped and BUN and creatinine levels rise. In this work, for the glycerol and glycerol-contrast medium groups, large increases in serum BUN and creatinine levels compared to the control group were observed. However, levosimendan, in both doses, decreased the serum BUN and creatinine concentrations (groups V–VIII). This fact suggests that levosimendan prevents contrast medium nephrotoxicity, especially since the decrease in BUN and creatinine was more prominent in the high dose levosimendan group (groups VII and VIII).

Various pathways have been proposed to explain the effects of levosimendan. Vascular resistance, arterial, and venous pressure all affect blood flow in the kidneys. Levosimendan corrects right ventricular function and reduces venous pressures [15] due to K+-ATP canals in afferent arterioles producing vasodilatation [16] or by mesangial cell contraction related to angiotensin-2 [17], or increasing the glomerular capillary surface area [18]. Additional advantageous paths are the drug’s preconditioning, anti-inflammatory, and anti-apoptotic effects on kidney tissue [19]. A study on the effect of levosimendan on postoperative kidney found that postoperative kidney dysfunction benefits from levosimendan administration [20]. Furthermore, a meta-analysis stated that the use of levosimendan is associated with a significant reduction in the requirement for renal replacement therapy in critically ill patients [21]. Another paper also pointed out that levosimendan has anti-inflammatory activity and decreases oxidative stress [12]. One study demonstrated the effects of levosimendan reducing oxidative stress and modulating pro-inflammatory cytokines in intestinal tissue [22]. SOD, GSH, MDA, TNF-α, NFK-β, and IL-6 considered in our study display the anti-oxidant and anti-inflammatory activities of levosimendan. In our study, levosimendan induced SOD and GSH and reduced MDA by its anti-oxidant effect and has reduced TNF-α, NFK-β, and IL-6 by its anti-inflammatory effect.

Oxidative stress is an important accompaniment of contrast medium nephrotoxicity. The formation of free radicals is accompanied by changes in SOD activity, GSH, and MDA levels [23]. Additionally, glycerol also decreases GSH [24]. A few drugs that induce nephrotoxicity also alter the level of these mediators. Thus, a study on propolis, a possible kidney-sparing drug, reported that it protects the kidney from free radicals and some adverse effects, through repairing the SOD activity, GSH, and MDA levels [23]. Another contrast nephrotoxicity study showed that ebselen administration decreased SOD activity [25]. Additionally, a cisplatin-induced nephrotoxicity model has shown the elevated MDA levels [26].

In our study, levosimendan restored levels of these biochemicals by vasodilation and anti-oxidative, anti-inflammatory effects. In addition, the MDA levels radically increased in glycerol and glycerol *plus* contrast medium groups according to the control group, whereas the SOD activity, GSH levels in glycerol, and glycerol *plus* contrast medium groups substantially diminished. However, levosimendan had the opposite effect on the levels of these chemicals: administration of levosimendan-improved SOD activity, GSH levels compared with glycerol, and glycerol *plus* contrast medium groups, with the best improvement seen in the high dose levosimendan group. These antioxidative consequences of levosimendan were revealed previously [12].

The inflammatory process influences nephrotoxicity. Macrophages raise the production of inflammatory cytokines along with the release of antioxidants [27]. It has been shown that the contrast medium injection causes increased levels of renal pro-inflammatory cytokines [24]. It has been demonstrated that TNF-α and IL-6 are also related to contrast nephrotoxicity [25, 28] and contrast administration increases NFK-β [29]. TNF-α is a pro-inflammatory cytokine and promotes various mediators related to tissue damage. In an animal model of nephrotoxicity, TNF-α plays a crucial role in the activation of the inflammatory response and the severity of damage [28]. IL-6 is also responsible for initiating the inflammatory response. Concerning our study, NFK-β plays an important role in numerous physiological processes, such as inflammatory and immune responses [30]. A factor known to activate NFK-β is an increase in intracellular calcium levels. Extracellular to intracellular Ca^++^ influx occurs during periods of oxidative stress and inflammation. Therefore, both Ca^++^ influx and oxidative stress increase NFK-β and cause damage to the kidney. Xu et al. [29] further showed that radiocontrast media administration increased NFK-β expression in glycerol and contrast medium groups. In our study, both doses of levosimendan were associated with significant downregulation of NFK-β, TNF-α, and Il-6 expressions. Since a stronger protective effect was seen with a higher dose of levosimendan, the findings of this study indicate that levosimendan has a potential dose-dependent nephroprotective effect by anti-inflammation.

Tubular necrosis, hyaline, and haemorrhagic casts have been found and interpreted as significant indicators in nephrotoxicity [14]. In our study, the histologic evaluation showed severe hyaline and haemorrhagic cast and moderate tubular necrosis for the glycerol group. Low-dose levosimendan administration relieved only hyaline casts. Although high dose levosimendan improved hyaline and haemorrhagic casts, there has been no effect on moderate tubular necrosis.

A limitation of the study was the limited number of rats used. Additionally, only two (not multiple) dose administrations have been applied. Furthermore, in our experiment, the levosimendan was administered intraperitoneally, whereas levosimendan for human use is administered intravenously; however, in rats, the bioavailability of intraperitoneal absorption is high, close to intravenous route. Additionally, multiple injections from the tail vein cause coagulation and occlusion. In this study, the contrast agent has been given from the tail vein.

In conclusion, this study showed that levosimendan has nephroprotective, antioxidant, and anti-inflammatory effects on contrast medium nephrotoxicity aggravated by glycerol in rats. Levosimendan provides this effect by decreasing oxidative stress and blocking the secretion of pro-inflammatory cytokines that trigger the inflammation in the kidney. Further clinical investigation is needed to better clarify the protective effects of levosimendan to protect from contrast medium nephrotoxicity.

## Data Availability

Data are available on request.

## Abbreviations

BUN: Blood urea nitrogen
cDNA: Complementary deoxyribonucleic acid
GSH: Glutathione
IL-6: Interleukin 6
MDA: Malondialdehyde
mRNA: Messenger ribonucleic acid
NFK-β: Nuclear factor kappa β
PCR: Polymerase chain reaction
RNA: Ribonucleic acid
SOD: Superoxide dismutase
TNF-α: Tumour necrosis factor α
